# Supplementary figures and images for: Preliminary analysis of New Zealand scampi (Metanephrops challengeri) diet using metabarcoding
Source: PeerJ. 2018 Sep 20;6:e5641. doi: 10.7717/peerj.5641 (PMC6151254; doi:10.7717/peerj.5641)

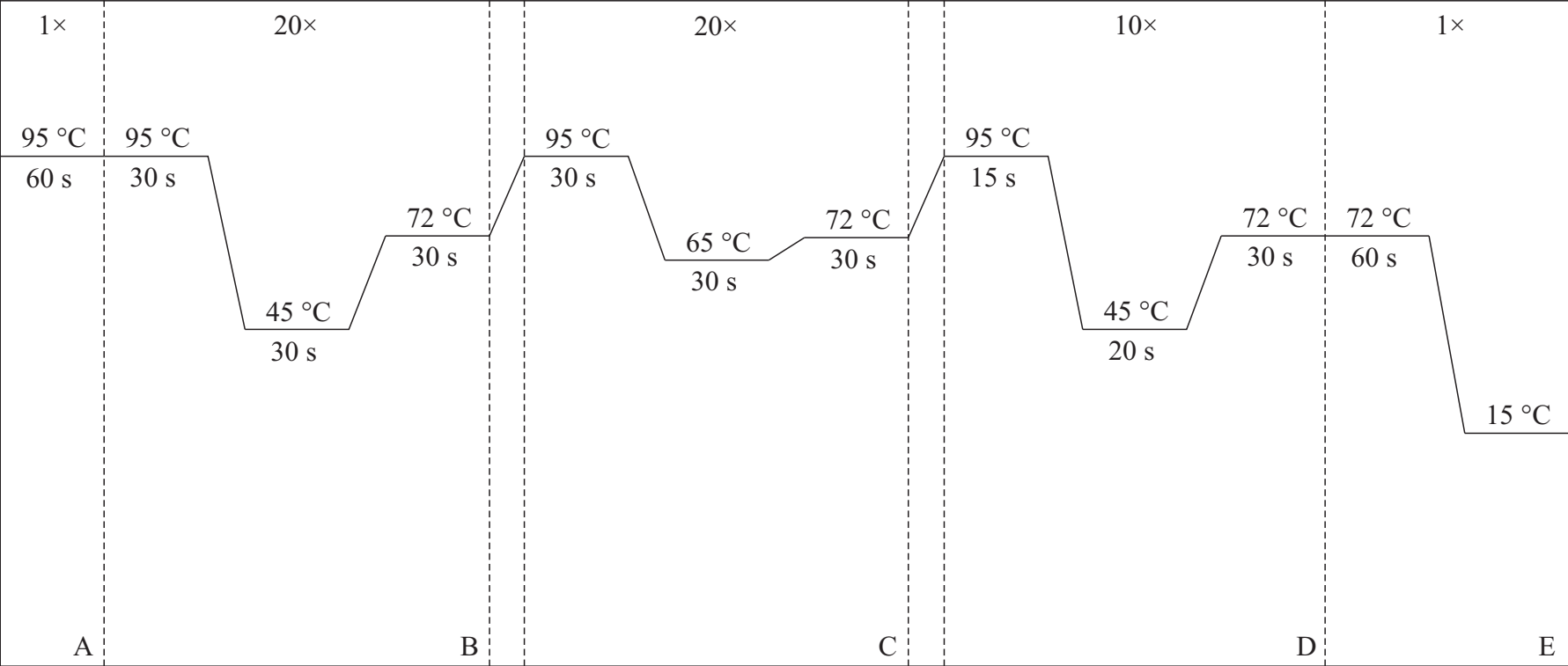

Supplement: Figure S1 — (A) Initial denaturation at 95 °C for 60 s. (B) 20 “touch-up” cycles, with each cycle having a denaturation step at 95 °C for 30 s, an annealing step starting at 45 °C for 30 s (each annealing cycle increases 1 °C every cycle) and a final extension step at 72 °C for 30 s. (C) 20 “touch-down” cycles, with each cycle having a denaturation step at 95 °C for 30 s, an annealing step starting at 65 °C for 30 s (each annealing cycle decreases 1 °C every cycle) and a final extension step at 72 °C for 30 s. (D) 10 cycles, with each cycle having a denaturation step at 95 °C for 15 s, an annealing step at 45 °C for 20 s and a final extension step at 72 °C for 30 s. (E) Final extension cycle at 72 °C for 60 s and holding at 15 °C. [file peerj-06-5641-s001.pdf]
