# Supplementary material for: Preliminary analysis of New Zealand scampi (Metanephrops challengeri) diet using metabarcoding
Source: PeerJ. 2018 Sep 20;6:e5641. doi: 10.7717/peerj.5641 (PMC6151254; doi:10.7717/peerj.5641)
Supplement: Table S1 — Nucleic acids have strong absorbance at 260 nm, which is also the wavelength where purines and pyrimidines peak. At 280 nm, proteins and phenolic compounds have a strong absorbance. Pure DNA should ideally be 1.8 for A260/A280 (Watts, 2014). [file peerj-06-5641-s002.docx]

| **DNA Extraction Kit** | **Individual** | **Concentration (ng µl-1)** | **A260**  **(10 mm)** | **A280**  **(10 mm)** | **A260/A280** | **DNA Visible on 0.8% Agarose Gel^[[1]](#endnote-1)^** |
| --- | --- | --- | --- | --- | --- | --- |
| DNeasy^®^ | 1 | 6.85 | 0.161 | 0.087 | 2.175 | Not visible |
| DNeasy^®^ | 2 | 3.00 | 0.050 | 0.012 | 2.727 | Not visible |
| DNeasy^®^ | 3 | 7.50 | 0.190 | 0.128 | 1.705 | Not visible |
| E.Z.N.A.^®^ | 1 | 1.80 | 0.041 | 0.011 | 6.000 | Not visible |
| E.Z.N.A.^®^ | 2 | 0.30 | 0.007 | -0.002 | -2.000 | Not visible |
| E.Z.N.A.^®^ | 3 | 0.00 | -0.034 | -0.030 | 1.211 | Not visible |
| Powerbiofilm^®^ | 1 | 5.75 | 0.182 | 0.118 | 2.255 | Not visible |
| Powerbiofilm^®^ | 2 | 4.50 | 0.191 | 0.160 | 1.525 | Not visible |
| Powerbiofilm^®^ | 3 | 4.40 | 0.141 | 0.092 | 2.256 | Not visible |
| Puregene^®^ | 1 | 86.20 | 5.038 | 4.767 | 1.187 | Visible |
| Puregene^®^ | 2 | 37.55 | 0.832 | 0.850 | 0.977 | Visible |
| Puregene^®^ | 3 | 25.00 | 0.578 | 0.509 | 1.160 | Visible |

1. Our primary consideration was concentration of high molecular weight DNA [↑](#endnote-ref-1)
