# Supplementary material for: Preliminary analysis of New Zealand scampi (Metanephrops challengeri) diet using metabarcoding
Source: PeerJ. 2018 Sep 20;6:e5641. doi: 10.7717/peerj.5641 (PMC6151254; doi:10.7717/peerj.5641)
Supplement: Table S2 — Reagent volumes and concentrations used in a 25 µl Platinum Taq reaction. [file peerj-06-5641-s003.docx]

| **Reagent** | **Volume (μl)** | **Concentration** | **Manufacturer** |
| --- | --- | --- | --- |
| Buffer | 2.50 | 10× | Invitrogen™, Thermo Fisher Scientific Inc., Massachusetts, USA |
| MgCl_2_ | 1.25 | 50 mM | Invitrogen™, Thermo Fisher Scientific Inc. |
| dNTPs | 0.20 | 20 mM | Bioline, London, UK |
| BSA | 2.00 | 1% | MP Biomedicals, California, USA |
| Platinum™ *Taq* | 0.05 | - | Invitrogen™, Thermo Fisher Scientific Inc. |
| Primer Forward | 0.40 | 10 μM | Integrated DNA Technologies Inc., Illinois, USA |
| Primer Reverse | 0.40 | 10 μM | Integrated DNA Technologies Inc. |
| DNA | 1.00 | - | - |
| UltraPure™ water | 17.20 | - | Invitrogen™, Thermo Fisher Scientific Inc. |
