# Supplementary material for: Preliminary analysis of New Zealand scampi (Metanephrops challengeri) diet using metabarcoding
Source: PeerJ. 2018 Sep 20;6:e5641. doi: 10.7717/peerj.5641 (PMC6151254; doi:10.7717/peerj.5641)
Supplement: Table S3 — Reagent volumes and concentrations used in a 25 µl Bioline reaction. [file peerj-06-5641-s004.docx]

| **Reagent** | **Volume (μl)** | **Concentration** | **Manufacturer** |
| --- | --- | --- | --- |
| MyTaq™ Red Mix | 12.5 | - | Bioline, London, UK |
| BSA | 2.0 | 1% | MP Biomedicals, California, USA |
| Primer Forward | 1.0 | 10 μM | Integrated DNA Technologies Inc., Illinois, USA |
| Primer Reverse | 1.0 | 10 μM | Integrated DNA Technologies Inc. |
| DNA | 1.0 | - | - |
| UltraPure™ water | 7.5 | - | Invitrogen™, Thermo Fisher Scientific Inc., Massachusetts, USA |
