# Supplementary material for: Preliminary analysis of New Zealand scampi (Metanephrops challengeri) diet using metabarcoding
Source: PeerJ. 2018 Sep 20;6:e5641. doi: 10.7717/peerj.5641 (PMC6151254; doi:10.7717/peerj.5641)
Supplement: Table S4 — Resulting totals for paired, merged and cleaned reads for COI and 18S. [file peerj-06-5641-s005.docx]

| **Sample** | **Individual** | **Digesta Source** | **PCR Reagent** | **COI Paired Reads** | **COI Merged reads** | **COI Cleaned Reads** | **18S Paired Reads** | **18S Merged reads** | **18S Cleaned Reads** |
| --- | --- | --- | --- | --- | --- | --- | --- | --- | --- |
| 1 | 70.9 | Hindgut | Bioline | 49651 | 47471 | 46094 | 23522 | 23313 | 17156 |
| 2 | 70.9 | Foregut | Bioline | 68150 | 34599 | 33669 | 64809 | 40483 | 33772 |
| 3 | 70.9 | Hindgut | Platinum *Taq* | 34081 | 663 | 627 | 77472 | 76562 | 62958 |
| 4 | 70.9 | Foregut | Platinum *Taq* | 97454 | 20587 | 20008 | 46361 | 39860 | 32369 |
| 5 | 70.2 | Hindgut | Bioline | 68530 | 50874 | 49446 | 31046 | 30673 | 24423 |
| 6 | 70.2 | Foregut | Bioline | 60917 | 7880 | 7629 | 71271 | 57689 | 47383 |
| 7 | 70.3 | Foregut | Bioline | 150414 | 39915 | 38883 | 69610 | 54609 | 45267 |
| 8 | Fro1 & Fro2 | Foregut | Bioline | NA | NA | NA | 105297 | 99788 | 81579 |
| 9 | Fro3 | Foregut and Hindgut | Bioline | 101356 | 38033 | 37080 | 32672 | 16780 | 13468 |
| 10 | DNA Negative | NA | Bioline | 146673 | 2231 | 2048 | 65664 | 5846 | 4796 |
