# Supplementary material for: Preliminary analysis of New Zealand scampi (Metanephrops challengeri) diet using metabarcoding
Source: PeerJ. 2018 Sep 20;6:e5641. doi: 10.7717/peerj.5641 (PMC6151254; doi:10.7717/peerj.5641)
Supplement: Table S5 — Nucleic acids have strong absorbance at 260 nm, which is also the wavelength where purines and pyrimidines peak. At 280 nm, proteins and phenolic compounds have a strong absorbance. Pure DNA should ideally be 1.8 for A260/A280 (Watts, 2014). [file peerj-06-5641-s006.docx]

| **Individual** | **Digesta Source** | **Concentration (ng µl^-1^)** | **A260 (10 mm)** | **A280 (10 mm)** | **A260/A280** |
| --- | --- | --- | --- | --- | --- |
| 70.2 | Hindgut | 57.15 | 3.509 | 3.121 | 1.514 |
| 70.2 | Foregut | 290.25 | 12.140 | 13.820 | 0.776 |
| 70.3 | Hindgut | 42.85 | 0.898 | 0.354 | 2.738 |
| 70.3 | Foregut | 11.85 | 0.255 | 0.168 | 1.580 |
| 70.9 | Hindgut | 29.90 | 0.615 | 0.348 | 1.807 |
| 70.9 | Foregut | 1.40 | 0.024 | 0.043 | 0.596 |
| Fro1 | Foregut | 124.85 | 3.804 | 3.352 | 1.221 |
| Fro2 | Foregut | 26.60 | 0.683 | 0.734 | 0.913 |
| Fro3 | Hindgut | 26.25 | 0.597 | 0.409 | 1.558 |
| Fro3 | Foregut | 3.10 | 0.087 | 0.102 | 0.805 |
