# Supplementary material for: Preliminary analysis of New Zealand scampi (Metanephrops challengeri) diet using metabarcoding
Source: PeerJ. 2018 Sep 20;6:e5641. doi: 10.7717/peerj.5641 (PMC6151254; doi:10.7717/peerj.5641)
Supplement: Table S6 — 18S genus reads were filtered from the 18S cleaned reads and then categorized into DNA negative reads, lobster and/or terrestrial reads and diet reads. [file peerj-06-5641-s007.docx]

| **Individuals** | **Cleaned Reads** | **Genus Reads** | **DNA Negative Reads** | **Lobster/Terrestrial Reads** | **Diet Reads** |
| --- | --- | --- | --- | --- | --- |
| 70.9 | 50928 | 50161 | 0 | 50151 | 10 |
| 70.2 | 71806 | 70841 | 0 | 70088 | 753 |
| 70.3 | 45267 | 44564 | 0 | 43299 | 1265 |
| Fro1 & Fro2 | 81579 | 81230 | 0 | 81230 | 0 |
| Fro3 | 13468 | 12988 | 0 | 12086 | 902 |
