# Supplementary material for: Preliminary analysis of New Zealand scampi (Metanephrops challengeri) diet using metabarcoding
Source: PeerJ. 2018 Sep 20;6:e5641. doi: 10.7717/peerj.5641 (PMC6151254; doi:10.7717/peerj.5641)
Supplement: Table S7 — The taxa are separated into their OTUs with their assigned taxonomic identity, hit counts, Midori RDP confidence levels, NCBI e-values and grouping. [file peerj-06-5641-s008.docx]

| **OTU** | **Taxonomic Identity** | **Hit Count** | **Midori RDP Confidence Level (%)** | **NCBI e-value** | **Grouping** |
| --- | --- | --- | --- | --- | --- |
| denovo70 | *Diadumene leucolena* | 8 | 100 | 1.35e-23 | Sea anemones |
| denovo7 | *Eimeria brasiliensis* | 568 | 100 | 1.30e-58 | Apicomplexa |
| denovo76 | *Eimeria brasiliensis* | 9 | 100 | 5.97e-67 | Apicomplexa |
| denovo108 | *Epinephelus epistictus* | 6 | 91 | 1.25e-83 | Fish |
| denovo5 | *Euchirella rostrata* | 1219 | 100 | 4.12e-148 | Copepods |
| denovo42 | *Funiculina quadrangularis* | 28 | 96 | 8.85e-150 | Sea pens |
| denovo15 | *Gammarus zaddachi* | 115 | 100 | 6.01e-62 | Amphipods |
| denovo109 | *Helicolenus barathri* | 5 | 100 | 4.03e-158 | Fish |
| denovo2 | *Hematodinium* sp. | 7363 | 100 | 1.95e-141 | Dinoflagellates |
| denovo67 | *Homola* sp. | 8 | 94 | 4.68e-58 | Crabs |
| denovo18 | *Hormosira banksii* | 98 | 100 | 5.92e-72 | Seaweed |
| denovo22 | *Hydrolagus novaezealandiae* | 60 | 100 | 4.06e-158 | Fish |
| denovo8 | *Laonice cirrata* | 486 | 100 | 1.68e-57 | Polychaeta |
| denovo29 | *Macruronus magellanicus* | 49 | 100 | 1.88e-161 | Fish |
| denovo32 | *Microphrys branchialis* | 19 | 92 | 7.50e-86 | Crabs |
| denovo28 | *Minutocellus polymorphus* | 45 | 100 | 3.39e-104 | Diatoms |
| denovo55 | *Neosarmatium fourmanoiri* | 10 | 95 | 3.49e-79 | Crabs |
| denovo35 | *Notophycis marginata* | 56 | 93 | 5.80e-87 | Fish |
| denovo101 | *Placoneis* sp. | 8 | 100 | 9.70e-85 | Diatoms |
| denovo24 | *Pleurosigma strigosum* | 69 | 100 | 1.22e-103 | Diatoms |
| denovo10 | *Seriolella punctata* | 359 | 100 | 2.41e-160 | Fish |
| denovo86 | *Seriolella punctata* | 5 | 100 | 3.24e-134 | Fish |
| denovo133 | *Skeletonema menzellii* | 8 | 100 | 1.62e-82 | Diatoms |
| denovo69 | *Thyrsites atun* | 10 | 100 | 3.13e-159 | Fish |
