# Supplementary material for: Preliminary analysis of New Zealand scampi (Metanephrops challengeri) diet using metabarcoding
Source: PeerJ. 2018 Sep 20;6:e5641. doi: 10.7717/peerj.5641 (PMC6151254; doi:10.7717/peerj.5641)
Supplement: Table S8 — COI OTUs, assigned sequences (genus and/or species level) and their GC percentage. [file peerj-06-5641-s009.docx]

| **OTU** | **COI Sequences** | **GC %** |
| --- | --- | --- |
| denovo10 | TAGCCGGAAACCTAGCCCACGCCGGAGCATCAGTTGACCTGACTATTTTTTCTCTCCACCTAGCAGGGATCTCCTCAATTCTAGGAGCCATTAATTTCATTACGACAATTATTAATATGAAGCCTGCAGCTATCTCCCAGTATCAAACACCCCTATTCGTTTGAGCCGTACTAATTACGGCCGTCCTACTTCTGTTATCTCTCCCTGTCCTCGCTGCTGGAATTACAATACTCCTTACAGACCGAAACCTAAACACAACCTTCTTTGATCCTGCAGGAGGAGGGGACCCAATCCTCTATCAACACCTCTTC | 46.62 |
| denovo101 | TTTATCAGGTATAACCGCTCATTCAGGAGGTTCTGTTGATTTGGCTATTTTTAGTCTTCATTTATCAGGAGCTGCTTCTATTTTAGGAGCGATAAATTTTATATGTACTATTACTAATATGAGATCTAAAAGCTTACCTTTCCATAGATTGCCTTTATTTGTTTGGTCGGTTTTAATTACTGCGTTCTTATTGTTACTATCTTTACCTGTTTTAGCAGGTGCTATCACTATGTTATTAACTGATCGTAATTTCAACACTACTTTTTTCGATCCAGCAGGAGGAGGAGATCCTGTGCTATACCAACATTTATTC | 34.19 |
| denovo108 | GGCAACCTCGCCCATGCAGGAGCATCCGTAGATTTAACTATTTTTTCTCTTCACTTAGCAGGAATTTCTTCTATCCCAGGGGCAATTAATTTTATTACAACCATTATTAATATAAAACCCCCCGCCATCTCCCAATACCAAACACCCTTATTTGTGTGAGCTGTTTTAATTACGGCCGTACTTCTCCTTCTCTCTCTTCCTGTCCTGGCTGCCGGCATCACTATGCTATTAACAGACCGAAACCTAAACACCACCTTCTTTGACCCGGCCGGAGGAGGGGACCCAATCCTCTATCAACACTTATT | 44.92 |
| denovo109 | TGGCTGGTAATTTAGCCCACGCGGGGGCATCCGTCGACCTGACAATTTTTTCGCTTCACCTAGCAGGTATTTCCTCAATCCTCGGGGCAATCAATTTTATTACCACAATTATTAACATGAAACCCCCAGCGATCTCTCAATACCAAACACCCCTGTTTGTGTGAGCTGTCCTAATTACCGCTGTTCTTCTCCTCCTCTCCCTACCCGTCCTTGCTGCAGGCATCACAATACTCCTTACGGACCGAAATCTTAATACCACCTTCTTCGACCCCGCTGGAGGAGGAGATCCTATCCTTTACCAACGCTTATT | 48.39 |
| denovo133 | TCAAGTGCAACTGCTCACTCTGGTGGGTCTGTGGATCTGGCCATATTCAGCCTGCACTTATCAGGTGCGTCTTCTATTTTAGGCGCTATTAACTTTATTTGTACAATTTTTAACATGCGAGTAAAGAGTTTATCTTTTCATAACCTTCCATTATTTGTGTGATCTGTCTTGATTACAGCATTTTTATTACTACTTTCGTTACCTGTTTTAGCGGGTGCTATTACTATGCTTTTAACAGATCGAAACTTCAATACGACATTTTTCGATCCTGCTGGAGGCGGAGATCCTGTATTATTTCAGCATCTTTTT | 37.86 |
| denovo15 | GCCCATAGAGGGGGAGCAGTAGATCTAGCAATTTTTTCTCTTCACCTTGCCGGGGCTCCCTCTATTTTAGGGGCAATTAATTTTATTACCACTGTGATCAATATACG-AGCAGCCGGAATATTTATAGACCGTGTCCCCTTATTTGTTTGGTCAGTTTTTATCACAGCTATTCTTTTGCTTCTGTCTCTACCTGTTTTAGCAGGGGCAATTACCATACTTCTCACTGACCGTAATATTAACACTTCATTTTTTGACCCTTTAGGAGGGGGGGACCCTATTCTCTACCAACA | 42.27 |
| denovo18 | GTAGATTTAGCTATTTTTAGTCTCCATTTATCAGGTGCGTCAAGTATTCTTGGTGCAATTAATTTCATTACAACTGTAATTAATATGCGCGCTCCAGGTATGAGTATGCACCGTTTACCTTTATTTGTATGGGCAGTTTTCATTACAGCATTCTTACTTTTATTATCACTTCCTGTATTTGCTGGAGCAATTACTATGTTATTAACAGATCGTAATTTTAATACTACGTTTTTTGATCCAGCAGGTGGTGGAGATCC | 35.02 |
| denovo2 | ATTAAGTACATCATTAATAAGTCTATCACCTATTGGAATTGATATTTTATTATATGGATTATTATTGTCAGGTATATCACCATGTCTAACATCTATTAATTTCATCGCTACAATTATAAATATGAGATGTTATAGTATGATATTATCGATTATGCCAGTATATACATGGTCACAAAATATTACAGGATTTCTAATGTTATTAACATTACCTATATTAACAGGAGCTCTTATAATGTCCTTAGCAGATCTTCATTATAATACAGTTTTCTTTAATCCAATATTTGGAGGCGATCCTGTACTTTATCAACATTTATTT | 26.58 |
| denovo22 | TCTAGCCGGTAATCTAGCACATGCCGGGGCATCTGTAGACCTAACCATTTTCTCCCTTCATTTAGCCGGGATTTCTTCAATTTTGGCCTCTATCAATTTCATTACTACAATTATTAACATAAAACCCCCATCAATCACCCAATATCAAACACCCCTATTTGTATGATCTATTTTAATTACTACAGTACTTCTCTTGCTATCTTTACCCGTTTTAGCAGCCGGTATTACAATATTACTAACAGATCGTAACCTAAATACAACATTCTTCGACCCAGCAGGAGGGGGGGATCCGATTTTATACCAGCACTTG | 39.86 |
| denovo24 | TTATCAAGTATCACAGCACACTCTGGAGGATCTGTTGATTTAGCAATTTTTAGTCTTCATTTATCTGGAGCTGCTTCTATTCTTGGAGCAATTAATTTTATTTGTACAATAACTAATATGAGAGCTAAAAGTTTACCATTCCATAGACTACCACTATTTGCTTGGTCAATTTTTATAACAGCTTTCTTATTACTATTATCATTACCAGTATTAGCAGGTGCGATTACTATGTTATTAACAGATCGTAACTTTAATACTACATTTTTTGA | 30.48 |
| denovo28 | CTTTCAAGTGGAACTTCTCACTCAGGTGGTGCTGTTGATCTAGCTATTTTCAGTTTACACCTTTCAGGAGCTCCATCAATCCTAGGAGCAATTAACTTTATATGTACCATCTTTAACATGAGAGTGAAAAGTTTGTCATTCCACAACTTACCTCTGTTTGTTTGGGCTGTTTTAATTACGGCTTTTTTACTATTGTTGTCTTTACCTGTTTTAGCAGGAGCAATAACAATGTTATTGACTGATAGAAATTTCAACACTACTTTCTTCGATCCTGCGGGAGGAGGAGATCCTGTGCTATACCAGCAT | 38.56 |
| denovo29 | CCTAGCCGGAAACCTCGCACATGCCGGAGCCTCCGTAGACCTGACTATCTTTTCTCTTCATTTAGCTGGTGTTTCATCTATTCTAGGGGCAATTAATTTTATTACCACTATTATTAACATGAAACCCCCAGCTATTTCACAATACCAAACACCATTGTTTGTATGAGCTGTATTAATTACCGCTGTCCTTCTTCTTTTATCACTTCCAGTTTTAGCAGCTGGTATTACAATGCTCTTAACTGACCGTAATCTTAACACTTCCTTCTTTGACCCTGCTGGAGGAGGGGACCCTATCCTCTACCAACACTTATTC | 41.85 |
| denovo32 | CTCTCGGCTGCTATTGTTCACGCAGGAGCCTCAGTTGATTTAGGTATTTTTTCTCTTCATTTAGCTGGTGTTTCATCAATCCTAGGTGCTGTTAATTTTATAACAACTGCAATTAATATACGAAGGAAAAGCATAACAATAGACCGAATACCTTTATTTGTCTGGTCAGTGTTTATTACAGCTATTCTGTCGCTACTCTCGCTTCCTGTTTTAGCCGGAGCAATTACTATACTCCTCACAGATCGAAATTTAAACACTTCATTTTTTGACCCAGCTGGGGGTGGAGACCCTGTTCTTTATCAACATTTATTT | 38.46 |
| denovo35 | TAGCAGGCAACCTCGCTCACGCGGGGGCCTCCGTTGATCTAACAATTTTTTCTCTCCACTTAGCAGGGATCTCTTCAATCCTGGGGGCAATTAACTTTATTACAACAATCATTAATATGAAACCCCCCGCTATCTCGCAGTACCAAACACCCCTATTTGTCTGAGCCGTCCTTATTACAGCAGTCCTCCTCCTCCTCTCCCTCCCTGTCTTAGCGGCCGGGATTACCATACTTTTGACGGACCGAAATTTAAATACTTCTTTCTTTGACCCTGCTGGAGGCGGGGACCCCATTCTTTACCAACATCTT | 48.38 |
| denovo42 | ATTAGCAAGTATTCAAGCTCATTCAGGAGGAGCAGTGGACATGGCCATATTCAGCTTACATCTAGCTGGGGTATCTTCCATTTTAAGTTCTATTAACTTTATTACTACTATAATTAACATGAGGATTCCTGGTATGAGTATGCATAGATTACCTCTATTCGTATGGTCTGTATTAGTTACTACTATATTGCTATTATTGTCTTTACCAGTATTAGCAGGTGCAATCACCATGTTATTGACAGACAGAAATTTTAATACGACATTCTTTGACCCCGCGGGAGGGGGGGATCCTATTTTATTCCAGCATCTATTC | 37.38 |
| denovo5 | ATTATCATCTAATATTGCTCATGCTGGAGGTAGGGTAGATTTTGCTATTTTTTCTTTACATTTAACAGGAGTTAGATCTATTCTTGGTGCAGTAAATTTTATTAGAACATTAGGAAATTTACGAGTATTTGAAATATTGTTAGATCGAATTCCACCATTTGCTTGATCAGTACTTATTACAGCTATTCTTTTATTATTATCATTACCTGTACTAGCTGGTGCAATTACTATATTACTAACTGATCGAAATTTAAATACAACATTTTATGATGTAGGGGGAGGCGGAGATCCTATTTTATATCAACACTTATTT | 30.03 |
| denovo55 | GCTGCTATTGCTCATGCAGGAGCCTCAGTTGATTTAGGTATTTTTTCTCTTCATTTGGCTGGTGTGTCATCAATTTTAGGGGCAGTTAATTTTATAACAACTGCAATCAATATACGAAGAAAAGGTATAACAATAGATCGAATACCTTTATTTGTTTGATCAGTATTTATTACAGCAATTTTATTACTTTTATCTCTTCCAGTATTGGCAGGAGCTATTACTATATTATTAACAGATCGTAATATTTCCACTTCTTTTTTTGACCCTTGTGGCGGAGGAGACCCTATCCCTTATCAACATTTATTT | 33.66 |
| denovo67 | TTTATCAGCTAG-AATCGCTCACAGAGGAGCTTCAGTCGATCTTAGAATTTTTTCATTACATTTAGCCGGAGGTTCTTCAATTATAGGAGCTATTAATTTTATTTCCAC---AATTATTA-ACCTCCACACTACTAG-AT-ACAATCCCCAAATTATACCTTTATTTATCTGATCAATTTTTATTACAGCAATTTTATTACTTTTATCTCTTCCAGTATTGGCAGGGGCTATTACTATACTCCTTACAGACCGAAATTTAAATACTTCATTTTTTGACCCCGCCGGAGGAGGTGACCCAGTTCTTTATCAACATTTATTC | 33.44 |
| denovo69 | CTCTCCGGGAACCTTGCCCACTCTGGAGCCTCCGTCGACCTGGCTATCTTTTCCCTGCACTTAGCAGGAATCTCATCAATTCTTGGGGCTATTAATTTTATCACGACCATCATTAATATGAAACCTCCAGCCATTTCTCAGTACCAAACACCCTTATTTGTCTGATCTGTTTTAATTACTGCAGTCCTCCTACTCCTATCACTTCCAGTCCTTGCTGCCGGTATTACTATGCTGCTGACAGACCGAAACCTTAATACAACTTTCTTTGACCCCTCCGGAGGAGGAGATCCAATTCTTTATCAGCACCTATTC | 45.51 |
| denovo7 | TAAGTACTTCACTAATGTCATTATCTCCTACCTCAGTAGATTTAATTGTAATAGGTCTAGCTTTTGCTGGAATATCTAGTCTATTATCCTCTATTAACTTTTTAAGTACAATAGCTGTATTAGGTGTAACTAATGGTTCTAAACCTTGGTGTTTATATACTTGGGCTATTGCATTTACTGCAATTATGTTAGTTGCTACATTACCAATATTAACCGGTGGTCTATTAATGTTAGTTCTAGATTTACATCTAAACACACAATTCTACGATGCATCATTTAATGGAGATCCTGTGTTATATCAACATCTATTC | 31.51 |
| denovo70 | TTAGCCTCCACTTGGCGGGGGCATCCTCAATTTTGGGAGCCATTAATTTTATCACGACCATTTTTAATATGCGGGCTCCCGGGATGATCATGCACAAGGTTCCGCTT-TTTGTGTGGGGAATTTTAATAACAGCATTTTTGCTGCTGTTGGCCGTTCCTGTTTTAGCGGGAGCTATCACCATGCTCTTAACAGATCGAGGG-TTTGGG-ACCAGTTTCTTTGACCCTGCAGGGGGGGGAGATCCCCTTCTCTTTCAACAT | 47.69 |
| denovo76 | TAAGTACTTCACTAATGTCATTGTCTCCTACCTCAGTAGATTTAATTGTAATAGGTCTAGC-ATTTGCTGGAATATCTAGTCTATTATCCTCTATTAATTTTTTGAGCACAATAGCTGTATTAGGTGTAACTAATGGTTCTAAACCTTGGTGTTTATTTACTTGGGCTATTGCATTTACTGCAATTATGTTAGTTGCTACATTACCAATTTTAACCGGTGGTCTATTGATGTTAGTTCTAGATTTACATCTAAACACACAATTCTATGATGCATCATTTAATGGAGATCCTGTGTTATATCAACATCTATTC | 32.05 |
| denovo8 | AATATTGCCCATGCTGGACCCTCCGTTGACATAGCAATTTTCTCTCTACATTTAGCAGGAATTTCCTCAATTTTAGGATCTATTAACTTTATCACCACAGTTATTAATATACGATGAAGGGGACTACAACTAGAGCGAATTCCCCTATTCGTCTGAGCCGTAAAAATTACTACCATCCTTCCCCTTCTCTCGCTCCCCGTTCTTGCAGGCGCCATTACTATGCTCTTAACAGATCGAAATCTCAACACTTCATTCTTTGACCCCGCAGGAGGAGGAGATCCAGTCCTATATCA-ACACTTATTT | 42.11 |
| denovo86 | GCTCATGCAGGAGCCTCAGTTGATTTAGGTATTTTTTCTCTCCACCTAGCAGGGATCTCCTCAATTCTAGGAGCCATTAATTTCATTACGACAATTATTAATATGAAGCCTGCAGCTATCTCCCAGTATCAAACACCCCTATTCGTTTGAGCCGTACTAATTATGGCCGTCCTACTTCTGTTATCTCTCCCTGTCCTCGCTGCTGGAATTACAATACTCCTTACAGACCGAAACCTAAACACAACCTTCTTTGACCCTGCAGGAGGAGGGGACCCAATCCTCTATCAACACCTCTTC | 45.12 |
