# Supplementary material for: Preliminary analysis of New Zealand scampi (Metanephrops challengeri) diet using metabarcoding
Source: PeerJ. 2018 Sep 20;6:e5641. doi: 10.7717/peerj.5641 (PMC6151254; doi:10.7717/peerj.5641)
Supplement: Table S9 — The taxa are separated into their OTUs with their assigned taxonomic identity, hit counts, SILVA RDP confidence levels, PR2 RDP confidence levels, NCBI e-values and grouping. [file peerj-06-5641-s010.docx]

| **OTU** | **Taxonomic Identity** | **Hit Count** | **SILVA RDP Confidence Level (%)** | **PR2 RDP Confidence Level (%)** | **NCBI**  **e-value** | **Grouping** |
| --- | --- | --- | --- | --- | --- | --- |
| denovo29 | *Cladococcus* | 43 | 89 | 100 | 0.0 | Rhizaria |
| denovo112 | *Cryothecomonas* sp. | 5 | 83 | 94 | 0.0 | Rhizaria |
| denovo45 | Dino-Group-I-Clade-1 X sp. strain | 23 | 100 | 96 | 0.0 | Dinoflagellates |
| denovo22 | Dino-Group-I-Clade-2 X sp. | 64 | 100 | 100 | 0.0 | Dinoflagellates |
| denovo3 | Dino-Group-I-Clade-2 X sp. | 419 | 100 | 100 | 0.0 | Dinoflagellates |
| denovo34 | Dino-Group-I-Clade-2 X sp. | 42 | 100 | 100 | 0.0 | Dinoflagellates |
| denovo38 | Dino-Group-I-Clade-2 X sp. | 48 | 84 | 100 | 0.0 | Dinoflagellates |
| denovo63 | Dino-Group-I-Clade-2 X sp. | 22 | 98 | 100 | 0.0 | Dinoflagellates |
| denovo7 | Dino-Group-I-Clade-2 X sp. | 199 | 88 | 100 | 0.0 | Dinoflagellates |
| denovo75 | Dino-Group-I-Clade-2 X sp. | 10 | 87 | 100 | 0.0 | Dinoflagellates |
| denovo65 | Dino-Group-I-Clade-3 X sp. | 12 | 99 | 100 | 0.0 | Dinoflagellates |
| denovo44 | Dino-Group-I-Clade-3 X sp. strain | 21 | 82 | 82 | 0.0 | Dinoflagellates |
| denovo56 | Dino-Group-I-Clade-4 X sp. | 27 | 81 | 99 | 0.0 | Dinoflagellates |
| denovo113 | Dino-Group-II XX sp. | 6 | 91 | 93 | 0.0 | Dinoflagellates |
| denovo24 | Dino-Group-II-Clade-15 X sp. | 42 | 100 | 100 | 0.0 | Dinoflagellates |
| denovo4 | *Duboscquella* | 261 | 100 | 96 | 0.0 | Dinoflagellates |
| denovo73 | *Duboscquella* | 12 | 100 | 100 | 0.0 | Dinoflagellates |
| denovo39 | *Ebria tripartita* | 43 | 100 | 100 | 0.0 | Rhizaria |
| denovo57 | *Eimeria percae* | 16 | 100 | 93 | 4.55e-154 | Apicomplexa |
| denovo124 | *Eimeria variabilis* | 5 | 87 | 83 | 0.0 | Apicomplexa |
| denovo26 | *Eimeria variabilis* | 49 | 91 | 82 | 0.0 | Apicomplexa |
| denovo13 | Eimeriidae sp. | 101 | 91 | 92 | 2.85e-111 | Apicomplexa |
| denovo105 | *Euchirella amoena* | 5 | 100 | 100 | 7.92e-152 | Copepods |
| denovo17 | *Euchirella amoena* | 77 | 100 | 100 | 0.0 | Copepods |
| denovo19 | *Euchirella amoena* | 71 | 100 | 100 | 3.61e-165 | Copepods |
| denovo2 | *Euchirella amoena* | 701 | 100 | 100 | 0.0 | Copepods |
| denovo33 | *Euchirella amoena* | 34 | 94 | 95 | 0.0 | Copepods |
| denovo46 | *Euchirella amoena* | 32 | 100 | 100 | 0.0 | Copepods |
| denovo66 | *Euchirella amoena* | 12 | 100 | 100 | 0.0 | Copepods |
| denovo94 | *Euchirella amoena* | 7 | 100 | 100 | 0.0 | Copepods |
| denovo119 | Filosa-Imbricatea Novel-clade-2 X | 5 | 99 | 100 | 0.0 | Rhizaria |
| denovo96 | *Goussia balatonica* | 9 | 83 | 87 | 4.70e-119 | Apicomplexa |
| denovo21 | *Ihlea racovitzai* | 70 | 100 | 100 | 0.0 | Tunicates |
| denovo32 | *Karlodinium* | 30 | 100 | 100 | 0.0 | Dinoflagellates |
| denovo28 | *Metridia pacifica* | 49 | 100 | 100 | 0.0 | Copepods |
| denovo30 | *Minidiscus trioculatus* | 36 | 80 | 100 | 0.0 | Diatoms |
| denovo108 | *Pycnococcus provasolii* | 5 | 97 | 86 | 0.0 | Prasinophytes |
| denovo117 | Pyrosomatidae sp. | 5 | 100 | 100 | 0.0 | Tunicates |
| denovo5 | *Selenidium* | 287 | 100 | 93 | 2.36e-161 | Apicomplexa |
| denovo51 | *Triastrum* | 20 | 96 | 100 | 0.0 | Rhizaria |
| denovo79 | *Umbellula* sp. | 5 | 91 | 92 | 0.0 | Sea pens |
