# Supplementary material for: Preliminary analysis of New Zealand scampi (Metanephrops challengeri) diet using metabarcoding
Source: PeerJ. 2018 Sep 20;6:e5641. doi: 10.7717/peerj.5641 (PMC6151254; doi:10.7717/peerj.5641)
Supplement: Table S10 — 18S OTUs, assigned sequences (genus and/or species level) and their GC percentage. [file peerj-06-5641-s011.docx]

| **OTU** | **18S Sequences** | **GC %** |
| --- | --- | --- |
| denovo105 | CGGGACCTTTACTTTGAGAAAAATAGAGTGTTTCAAGCAGGCTTTACGCCTTGAATACTGCAGCATGGAATAATAAGATAGGACTTTGGTTCTATTTTGTTGGTTA-TAGGACTAAAGTAATGATTAATAGGGACAGTTGGGGGCATTCGTATTCAACAGTCAGAGGTGAAATTCTTAGATTTGTTAAAGACGAACTACTGCGAAGGCATTTGCCAAGGATGTTTTCACTAATCAAGAACGACAGTAGGGGGTT | 38.98 |
| denovo108 | AGCCGCGGTAATTCCAGCTCCAATAGCGTATATTTAAGTTGTTGCGGTTAAAAAGCTCGTAGTTGGATTTCGGGCGTGGAGGTTCGGTCCGCCGTTTCGGTGTGCACTGTCTTTCTGCGTCTTTCTGTCGGGGACGCGCTTCTGGCCTTAATTGGCTGGGACGTGGATTCGACGAGGTTACTTTGAAAAAATTAGAGTGTTCAAAGCAAGCTAAAGCTCTGAATACATTAGCATGGAATAACGCGAGAGGACTCGGTCCCTATTTTGTTGGTCTTCAAGGATGGAGTAATGATTAAGAGGGACAGTTGGGGGCATTCGTATTTCATTGCTAGAGGTGAAATTCTTGGATTTATGAAAGACGAACTTCTGCGAAAGCATTTGCCAAGGATGTTTTCATTGATCAAGAACGAAAGTTGGGGGCTCG | 45.99 |
| denovo112 | AGCCGCGGTAATTCCAGCTCCAAGAGCGTATATTAAAGTTGTTGCAGTTAAAAAGCTCGTAGTTGGATTTCTGGTATAACGCGCCTGGCCCGCTTTTGTGAGTGCCGGTTGCACGTTTGCCATCCTTCTAGAAACCGTTTCTACCCGGGCAACTGGGCCGGACTCGTGATCTAGATCTTTTACTTTGAAAAAATTAGAGTGTTTAAAGCAGGCTTTTGCTATGAATACATTAGCATGGAATAATAATCTAGGACTTCGGTTCTATTTTGTTGGTTTCTAGTACTGAAGTAATGATTGATAGGGATAGTTGGGGGTGCTAGTATTGGCCGGCCAGAGGTGAAATTCTTGGATTCGGTCAAGACTAACTTATGCGAAAGCATTCACCAAGGATGTCTTCTTTAATCAAGAACGAAAGTTGGGGGATCG | 43.43 |
| denovo113 | AGCCGCGGTAATTCCAGCTCCAATAGCGTATATTAAAGTTGTTGCGGTTAAAAAGCTCGTAGTTGGATTTCTGTCGAGGACGTCCGGTCCATCTTTCGGGTGAGTATCTGGTACGGCCTTGGCATCCTCTTGGAGAACTACGCTGCACTTGACTGTGTGGTTAGGGATTCAAGACTTTTACTTTGAGGAAATTAGAGTGTTCACGGCAGGCAAACGTCTGAATACATTAGCATGGAATAATAATATAGGACCTTGGTTCTATTTTGTTGGTTTCTAGGACTAAGGTAATGATTAATAGGGATAATTGGGGGCATTCGTATTAACGCGTCAGAGGTGGAATTCTTGGATTGCGTTACGACGAACTACTGCGAAAGCATTTGCCAAGGATGTTTTCATTGATCAAGAACGAAAGTTAGGGGATCG | 43.50 |
| denovo117 | AGCCGCGGTAATTCCAGCTCCAAAAGTGTATATTTAAGTTGTTGCGGTTAAAAAGCTCGTAGTTGGATTCCGGGCTCGGGCGGTCGGTCCGTCGCAAGGCGTGTACTGGCCTGTCCCGACCTCACCTTCGGTGCTCCGTCGGTGCTCTTGACTGAGTGTCGCGCGGTGGCCGGAACGTTTACCTTGAAAAAATTAGAGTGTTCAACGCAGGCTTTCAAGCTTGCATAATGGTGCATGGAATAATGAAATAGGACCTCGGTTCTATTTTGTTGGTTTTCGGAGCGCGAGGTAATGATTAAGAGGAACGGACGGGGGCATCCGTACTGTGCCGTTAGAGGTGAAATTCTTGGATCGGCGCAAGACGAACGACTGCGAAAGCATTTGCCAAGAATGTTTTCTTTAATCAAGAGCGAAAGTCAGAGGTTCG | 50.35 |
| denovo119 | AGCCGCGGTAATTCCAGCTCCAGTAGCGTATACTAATGTTGCTGCAGTTAAAAAGCTCGTAGTTGGATTTCTGGTTGATAATGTTGGTCCGAGTGTTCCTCCGGGAACCTTGAGTATCGATTCTTATCTACCATCCTATCAGTGTTGGTTCCTTCTGGT-TCCAGTTCTGGTACTTTTACTTTGAAAAAATTAGAGTGTTTAAAGCAAGCTTGCGCGATGAATACATTAGCATGGAATAATAATATAGGACTACGGGTTCTATTTTGTTGGTTTCTAGACCTGAAGTAATGATTGATAGGGATAGTTGGGGGTGCTAGTATTGAGAGGCCAGAGGTGAAATTCTTTGATTCTCTCAAGACTAACCTATGCGAAAGCATTCACCAAGGATGTCTTCTTTAATCAAGAACGAAAGTTGGGGGATCG | 41.04 |
| denovo124 | AGCCGCGGTAATTCCAGCTCCAATAGCGTATATTAGAGTTGTTGCAGTTAAAAAGCTCGTAGTTGGATTTCTGTTGGGGGCAGCTCGTCCGCCTTTTGGTGTGCACAAGTCATCCCTAGCATTTTTCTGGTTCCCCTTTGCACTTTATTGTGCTTTGGTTCTCCAGAACGTTTACTTTGAGAAAAATAGAGTGTTTCAAGCAGGCTTTTGCCTTGAATACTGCAGCATGGAATAATAAGATAGGACTTTGGCCCTATTTTGTTGGTTCTAGGACTAAAGTAATGATTAATAGGGACAGTTGGGGGCATTCGTATTTAACTGTCAGAGGTGAAATTCTTAGATTTGTTAAAGACGCACTACTGCGAAAGCATTTGCCAAGGATGTTTTCATTAATCAAGAACGAAAGT | 41.03 |
| denovo13 | CGGGACCTTTACTTTGAGAAAAATAGAGTGTTTCAAGCAGGCTTTACGCCTTGAATACTGCAGCATGGAATAATAAGATAGGACTTTGGTTCTATTTTGTTGGTTA-TAGGACTAAAGTAATGATTAATAGGGACAGTTGGGGGCATTCGTATTCAACAGTCAGAGGTGAAATTCTTAGATTTGTTAAAGACGAACTACTGCGAAGGCATTTGCCAAGGATGTTTTCACTAATCAAGAACGACAGTAGGGGGTT | 38.98 |
| denovo17 | AGCCGCGGTAATTCCAGCTCCAATAGCGTATATTAAAGTTGTTGTGGTTAAAAAGCTCGTAGTTGGATCTCGGCGGTCTGAGGGCGGTTCACTTGCTAGTGCTACTGCCCTATAGACCGTAGTTTTGCCAGAGGTCTCAGGGTGCTCTTAATCGAGTGTCTTGTGATGCTGGCAGGGTTACTTTGAAAAAATTAGAGTGCTCAAAGCAGGCTTTTACGCCTGAATATTCGTGCATGGAATAATAGAATAGGAAGTTGTTTCTATTTTGTTGGTTTTCGGAAATCGA-------------AGGGACAGTCGGGGGCATTTGTATTCAAACGACAGAGGTGAAATTCTTGGACCGTTTGAAGACAAACTACTGCGAAAGCATTTGCCAAGAATGTTTTCATTAATCAAGAACGAAAGTTAGAGGTTCG | 42.02 |
| denovo19 | AGCCGCGGTAATTCCAGCTCCAATAGCGTATATTAAAGTTGTTGTGGTTAAAAAGCTCGTAGTTGGATCTCGGCGGTCTGAGGGCGGTTCACTTACTAGTGCTACTGCCCTATAGACCGTAGTTTTGCCAGAGGTCTCAGGGTGCTCTTAATCGAGTGTCTTGTGATGCTGGCAGGTTTACTTTGAAAAAATTAGAGTGCTCAGAGCAGGCTATTTGAATGGCCCGAATGGTGATGCATGGAATAATGGAATAGGACCTCGGTTCTATTTTGTTGGTTTTCGGAACCAGAGGTAATGACTAATCGGAACAGGCGGGGGCATTCGTATTGCGACGCTAGAGGTGAAATTCTTGGACCGTCGCAAGACGAACTACTGCGAAAGCATTTGCCAAGGATGTTTTCATTAATCAAGAACGAAAGTTAGAGGTTCG | 46.05 |
| denovo2 | AGCCGCGGTAATTCCAGCTCCAATAGCGTATATTAAAGTTGTTGTGGTTAAAAAGCTCGTAGTTGGATCTCGGCGGTCTGAGGGCGGTTCACTTGCTAGTGCTACTGCCCTATAGGCCGTAGTTTTGCCAGAGGTCTCAGGGTGCTCTTAATCGAGTGTCTTGTGATGCTGGCAGGTTTACTTTGAAAAAATTAGAGTGCTCAGAGCAGGCTTTTACGCCTGAATATTCGTGCATGGAATAACAGAATAGGAAGTCGTTTCTATTTTGTTGGTTTTCGGAAATCGACTTAATGATTAATAGGGACAGTCGGGGGCATTTGTATTCAAACGACAGAGGTGAAATTCTTGGACCGTTTGAAGACAAACTACTGCGAAAGCATTTGCCAAGAATGTTTTCATTAATCAAGAACGAAAGTTAGAGGTTCG | 43.19 |
| denovo21 | AGCCGCGGTAATTCCAGCTCCAAAAGTGTATATTTAAGTTGTTGCGGTTGAAAAGCTCGTAGTCGGATTTCAGGGCTGGCGTTTCGGTCCCTCGTAAGAGTGGTACTGAAGCGTCGCCCTCACCTCCGGTGTCACGCCGGTGCTCTTAACTGAGTGCCGGTGCGGTCCGGAAATTTTACCTTGAAAAAATTAGAGTGTTTATAGCAGGCGTTTTGCTTGTATAATGTTGCATGGAATAATGGAATAGGACCTCGGTTCTATTTTGTTGGTTTTCGGAGCGCGAGGTAATGACCAATAGGAACGGACGGGGGCATCCGTACTGTGCCGTTAGAGGTGAAATTCTTGGATCGGCGCAAGACGAACAACTGCGAAAGCATTTGCCAAGAATGTTTTCTTTGATCAAGAGCGGAAGTCAGAGGTTCG | 48.46 |
| denovo22 | AGCCGCGGTAATTCCAGCTCCAATAGCGTATATTAAAGTTGTTGCGGTTAAAACGCTCGTAGTTGGGTTTCTGTCAGGAACAACTGGTCTGTCCTGAGTGTCGAGCACTAGTTCGGCTCCTGGCATCTTCTTGGGGAACGGGTGCGCACTTCACTGTGTGGCCTGGAAACCTTGACGTTTACTTTGAGGAAATGAGAGTGCTTCAAGCGGGTGTCCGCCACGAATACGTTAGCATGGAATAATAGTATTGGACCTCGGATCACAGCTGTTGGTTGCTGGAATCGGGGTGAGGGTAAACAGGGACAGTTGGGGGCATTCGTATTTAACTGTCAGAGGTGAAATTCTTGGATTTGTTAAAGACGGACTACTGCGAAAGCATTTGCCAAGGATGTTTTCATTGATCAAGAACGAAAGTTAGGGGA | 47.87 |
| denovo24 | AGCCGCGGTAATTCCAGCTCCAATAGCGTATATTAAAGTTGTTGCGGTTAAAAAGCTCGTAGTTGGATTTCTGTTGAGAGTGGCCAGTCCGCACTTAGTGCGAGTATTTGGTTCAGTCTCGGCATCCTCCAGGGGAGCGTACTGTACTTGATTGTATGGTGCGCAATTCTGGACTTTTACTTTGAGGAAATTAGAGTGTTCACGGCAGGCATTTGCCTTGAATATGTTAGCATGGAATAATAATATAAGACCTTGGTCTTATTTTGTTGGTTCCTAGGAGCAGGGTAATGATTAATAGGGATAATTGGGGGTATTCGAATTAAGGCGTCAGAGGTGGAATTCTTGGATTGCCTTACGACGAACTACTGCGAAAGCATTTACCAAGGATGTTTTCATTGATCAAGAACGAAAGTCAGGGGATCG | 43.26 |
| denovo26 | AGCCGCGGTAATTCCAGCTCCAATAGCGTATATTAGAGTTGTTGCAGTTAAGAAGCTCGTAGTTGGATTTCTGTTGGGGGCAGCTCGTCCGCCTTTTGGTGTGCACAAGTCATCCCTAGCATTTTTCTGGTTCCCCTTTGCACTTTATTGTGCTTTGGTTCTCCAGAACGTTTACTTTGAGAAAAATAGAGTGTTTCAAGCAGGCTTTTGCCTTGAATACTGCAGCATGGAATAATAAGATAGGACTTTGGCCCTATTTTGTTGGTTCTAGGACTAAAGTAATGATTAATAGGGACAGTTGGGGGCATTCGTATTTAACTGTCAGAGGTGAAATTCTTAGATTTGTTAAAGACGCACTACTGCGAAAGCATTTGCCAAGGATGTTTTCATTAATCAAGAACGACAGTAGGGGGTTTG | 41.97 |
| denovo28 | AGCCGCGGTAATTCCAGCTCCAATAGCGTATATTAAAGTTGTTGCGGTTAAAAAGCTCGTAGTTGGATCTCGGCAAGTAGAGGGCGGTATGCTTCGTAGCATTACTGCCCTATTACTTGTAGTTTTGCCAGAGGTTCCTGGGTGCTCTTAATCGAGTGTCTAGGGATGCTGGCCGGTTTACTTTGAAAAAATTAGAGTGCTCAAAGCAGGCTATTACGCTTGAATATTCGTGCATGGAATAATAGAATAGGAAGTTGTTTCTATTTTGTTGGTTTTCGGAAATCGACTTAATGATTAATAGGGACAGTCGGGGGCATTTGTATTCAAACGACAGAGGTGAAATTCTTGGACCGTTTGAAGACAAACTACTGCGAAAGCATTTGCCAAGGATGTTTTCATTAATCAAGAACGAAAGTTAGAGGTTCG | 41.78 |
| denovo29 | AGCCGCGGTAATTCCAGCTCCAATAGCGTATACTAAAGTTGTTGCAGTTAAAAAGCTCGTAGTTGGATTTCTGTGGGGACGGAGCGGCCGGCCGCACATCGCGGTTCGAGCTAGCGCTTTCCCTACATCCTGCCGCGAACCGTGCTACCATTAGTTTGGTGGCGCCGGGGATCGGCTCGATTACTTTGAGAAAAATAGAGTGTTCAAAGCAGGCCACACGCCTTGAATAAGTTAGCATGGAATAATAACTAAGGACTTCGGTTCTATTTTGTTGGTGTTCGGGACTGAAGTAATGATTGATAGGGACAGTTGGGGCGGTCAGTATTCCAAAGCGAGAGGTGAAATTCTTAGACCTTCGGAAGACTAACAGCTGTGAAAGCGTTCCGCAAGGATGTTCCCTTTGATCAAGAACGAAAGTTGGGGGATCG | 49.07 |
| denovo3 | CGGTAATTCCAGCTCCAATAGCGTATATTAAAGTTGTTGCGGTTAAAACGCTCGTAGTTGGGTTTCTGTCAGGAACAACTGGTCTGTCCCATGTGTCGAGCACTAGTTCGGCTCCTGGCATCTTCTTGGGGAACGGGTGCGTACTTCACTGTATGGCCTGGAAACCTTGACGTTTACTTTGAGGAAATGAGAGTGCTTCAAGCGGGTGTCCGCCACGAATACGTTAGCATGGAATAATAGTATTGGACCTCGGATCATAGCTGTTGGTTGCTGGAATCGGGGTGAGGGTAAACAGGGACAGTTGGGGGCATTCGTATTTAACTGTCAGAGGTGAAATTCTTGGATTTGTTAAAGACGGACTACTGCGAAAGCATTTGCCAAGGATGTTTTCATTGATCAAGAACGAAAGTCAGGGGATCG | 47.14 |
| denovo30 | AGCCGCGGTAATTCCAGCTCCAATAGCGTATATTAAAGTTGTTGCAGTTAAAAAGCTCGTAGTTGGATTTCTGGCAGGAGCGACCGGTCACACACTCTGTGTGTGAACTTGCGTTGTCTCTGGCCATCCTTGGGAAGATCCTGTTTGGCATTAAGTTGTCGGGCAGGGGATACCCATCGTTTACTGTGAAAAAATTAGAGTGTTTAAAGCAGGCTTATGCCGTTGAATATATTAGCATGGAATAATAAGATAGGACTTCGGAACTATTTTGTTGGTTTGCGTTACGAAGTAATGATTAATAGGGACAGTTGGGGGTATTCGTATTTCGTTGTCAGAGGTGAAATTCTTGGATTTCCGAAAGACGAACTACTGCGAAAGCATTTACCAAGGATGTTTTCATTAATCAAGAACGAAAGTTAGGGGATCG | 42.15 |
| denovo32 | AGCCGCGGTAATTCCAGCTCCAATAGCGTATATTAAAGTTGTTGCGGTTAAAAAGCTCGTAGTTGGAGTTCTGCCAGGTGCCGCCTGTCCGCCCCAGTGGTGAGTACGTGGCGCGCATTTGGCCCTTTCAAGGGGAGCGTATCTGCACTTTATTGTGTGGTGCGGGATCCTTGACTTTTACTTTGAGGAAATAGGAGTGTTCCAAGCAGGCTCTCGTCGTGCACAGCTCAGCATGGAATAATAGCATTGGACCTCGATTCTAAGCTGTTGGTTGCCAGAAGCGAGGTAATGATGAAGAGGGATAGTTGGGGGCATTCGTATTTAACTGTCAGAGGTGAAATTCTTGGATTTGTTAAAGACGGACTACTGCGAAAGCATCTGCCATGGATGTTTTCATTGATCAAGAACGAAAGTTAGGGGATCG | 48.11 |
| denovo33 | AGCCGCGGTAATTCCAGCTCCAATAGCGTATATTAAAGTTGTTGTGGTTAAAAAGCTCGTAGTTGGATCTCGGCGGTCTGAGGGCGGTTCACTTGCTAGTGCTACTGCCCTACAGACCGTAGTTTTGCCAGAGGTATCAGGGTGCTCTTAATCGAGTGTCTTGTGATGCTGGCAGGTTTACTTTGAAAAAATTAGAGTGCTCAAAGCAGGCTTTTACGCCTGAATATTCGTGCATGGAATAATAGAATAGGACCTCGGTTCTATTTTGTTGGTTTTCGGAACCAGAGGTAATGACTAATCGGAACAGGCGGGGGCATTCGTATTGCGACGCTAGAGGTGAAATTCTTGGACCGTCGCAAGACGAACTACTGCGAAAGCATTTGCCAAGGATGTTTTCATTAATCAAGAACGAAAGTTAGAGGTTCG | 45.54 |
| denovo34 | AGCCGCGGTAATTCCAGCTCCAATAGCGTATATTAAAGTTGTTGCGGTTAAAACGCTCGTAGTTGGGTTTCTGTCAGGAACAACCGGTCTGTCCTGAGTGTCAAGTCACTGGTTCGGCACCTGCCATCTTCTTGGGGAACGGGTGCGCACTTCACTGTGCGACCTGGAAACCTTGACGTTTACTTTGAGGAAATGAGAGTGCTTCAAGCGGGTGTTCGCCACGAATATGTTAGCATGGAATAATAGTATTGGACCTCGGATCACAGCTGTTGGTTGCTGGAATCGGGGTGAGGGTAAACAGGGACAGTTGGGGGCATTCGTATTTAACTGTCAGAGGTGAAATTCTTGGATTTGTTAAAGACGGACTACTGCGAAAGCATTTGCCAAGGATGTTTTCATTGATCAAGAACGAAAGTTAGGGGATCG | 47.65 |
| denovo38 | AGCCGCGGTAATTCCAGCTCCAATAGCGTATATTAAAGTTGTTGCGGTTAAAACGCTCGTAGTTGGGTTTCTGTCAGGAACAACCGGTCTGTCCTGAGTGTCAAGTCACTGGTTCGGAACTGGCATTTTCTTGGGGAACGGGTGCGCACTTTACTGTGCGACCTGGAAACCTTGACGTTTACTTTGAGGAAATGAGAGTGCTTCAAGCGGGTGTTCGCCACGAATATGTTAGCATGGAATAATAGTATTGGACCTCGGATCACAGCTGTTGGTTGCTGGAATCGGGGTGAGGGTAAACAGGGACAGTTGGGGGCATTCGTATTTAACTGTCAGAGGTGAAATTCTTGGATTTGTTAAAGACGGACTACTGCGAAAGCATTTGCCAAGGATGTTTTCATTGATCAAGAACGAAAGTTAGGGGATCG | 46.82 |
| denovo39 | AGCCGCGGTAATTCCAGCTCCAATAGCGTATATTAAAGTTGTTGCAGTTAAAAAGCTCGTAGTTGGATTTCTGGTTTGATGCCCTGGCCCGCTGCTATTTTGGTAGTGTGTGCCGGTTGTGCGTCTGCCATCCTTCTAGGGAACGTTCCTTCTCTTCACTGAGCCGGGTTCGTGATCTAGATCGTTTGCTTTGAAAAAATTAGAGTGTTTAAAGCAGGCTTTTGCTATGAATACATTAGCATGGAATAATAATCTAGGACTTCGGTGCTATTTTGTTGGTTTCTAGAACTGAAGTAATGATTGATAGGGATAGTTGGGGGTGCTAGTATTGAGCGGCCAGAGGTGAAATTCTTGGATTCGCTCAAGACTAACTTATGCGAAAGCATTCACCAAGGATGTCTTCTTTAATCAAGAACGAAAGTTGGGGGATCG | 43.29 |
| denovo4 | AGCCGCGGTAATTCCAGCTCCAATAGCGTATATTAAAATTGTTGCGGTTAAAACGCTCGTAGTTGGATATCTGTGAGCGGGTTCCGGTCCTTCCCAGTGAAGATTACACGGAACTCTGCTCGCATTTGTTCAGGGAGGGTGGATGCACTTCACTGTGTGACCACATGATCTGAATTTTTACTTTGAGGAAATGAGAGTGTTCCAAGCAGGCAATTGCCGTGAATATGATAGCATGGAATAATAGCACAGGACTCTTGGTTCAATGCTGTTGGTTTGTTGAACTAGAGTAATCACAATAAGGATAGTTGGGGGTATTCGTATTTAACTGTCAGAGGTGAAATTCTTGGATTTTTTAAAGACGAACAATTGCGAAGGCATCTACCCAGGATGTTTTTGTTGATCAAGAACGAAAGTTAGGGGATCG | 42.69 |
| denovo44 | AGCCGCGGTAATTCCAGCTCCAATAGCGTATATTAAAATTGTTGCGGTTAAAACACTCGTAGTTGGATATTTGTTGAGAACTGCCGGTCTACCCCAGTGGTCAGCACACGGTTTGTTCTCGGCACTTTCCTGGGGAGAGTGTTTGCACTTCACTGTGTGGCACATGATCCAGAACTTTTACTTTGAGGAAATGAGAGTGTTTCAAGCAGGCGCCTGTCGTGAATACGTTAGCATGGAATAATAGCATGGGACCGCGGTCTGAAGCTGTTGGTTTTTGGGCCGTGGTAATGAAAATAGGGATAGTTGGGGGTATTCGTATTTAACTGTCAGAGGTGAAATTCTTGGATTTTTTAAAGACGGACTACTGCGAAAGCATCTGCCCAGGATGTTTTTATTGATCAAGAACGAAAGTTAGGGGATC | 44.89 |
| denovo45 | AGCCGCGGTAATTCCAGCTCCAATAGCGTATATTAAAGTTGTTGCGGTTAAAAAGCTCGTAGTTGGAGTTCTGCCAGGTGACGCTCGTCCGCCCAAGTGGTGTGTACAGGGTGTGCATCTGGCCCTTTCAAGGGGAACGTGTCTGCACTTCATTGTGTGGTGCGAGATCCTTGACTTTTACTTTGAGGAAATAAGAGCGTTCCAAGCAGGCTCTCGTCGTGCATAGCTCAGCATGGAATAATAGCATTGGACTTCGTTTCTACGCTGTTGGTTGCAAGAAGCGAGGTAATGATGAAGAGGGATAGTTGGGGGCATTCGTATTTAACTGTCAGAGGTGAAATTCTTGGATTTGTTAAAGACGGACTACTGCGAAAGCATCTGCCATGGATGTTTTCATTGATCAAGAACGAAAGTTAGGGGATCG | 46.93 |
| denovo46 | AGCCGCGGTAATTCCAGCTCCAATAGCGTATATTAAAGTTGTTGTGGTTAAAAAGCTCGTAGTTGGATCTCGGCGGTCTGAGGGCGGTTCACTTGCTAGTGCTACTGCCCTATAGACCGTAGTTTTGCCAGAGGTCTCAGGGTGCTCTTAATCGAGTGTCTTGTGATGCTGGCAGGGTTACTTTGAAAAAATTAGAGTGCTCAAAGCAGGCTTTTACGCCTGAATATTCGTGCATGGAATAATAGAATAGGAAGTCGTTTCTATTTTGTTGGTTTTCGGAACCAGAGGTAATGACTAATCGGAACAGGCGGGGGCATTCGTATTGCGACGCTAGAGGTGAAATTCTTGGACCGTCGCAAGACGAACTACTGCGAAAGCATTTGCCAAGGATGCTTTCATTAATCAAGAACGAAAGTTAGAGGTTCG | 45.54 |
| denovo5 | CGGTAATTCCAGCTCCAATAGCGTATATTAAAGTTGCTGCAGTTAAAACGCTCGTAGTTGTTGTCCAACTATACGCTTCATTGCATTTTGCTATTGAAGTACATAGTTTAGCTGAGACTTTGTTTCGGCCAAAACCTTGAGGAAAGATTAGTGTTCCAAGCAGCTAACTTTGTCCTCCCCAGCATGGTATTCAACTCAATCAGAGGAGGGTTGCCGAGCCCTCATCAAACTAAATAGAAATTCAAGGAGGTATACGTACTTTAATGTCAGAGGTGAAATTCCTAGATTTTTAAAAGACGGTCAGTTGCGAAAGCGTTCACCAAGCGACTCTTCTTTGATCAAGAACGAAAGTTGGGGGATTG | 41.44 |
| denovo51 | AGCCGCGGTAATTCCAGCTCCAATAGCGTATACTAAAGTTGTTGCAGTTAAAAAGCTCGTAGTTGGATTTCTGCGCGGGCGGGGCGCCCGCCGGCCTAAG-GTCGGGTTGGGCAGCAACGCCCGCGCATCCTGCCGCGAACCACGCTACCATTAGTTTGGTGGCGTCTTGGGGACCGGCTCGTTTACTTTGAGAAAAATAGAGTGTTCAAAGCAGGCAACTCGCCTTGAATAATGTAGCATGGAATAATGATAAAGGACTTTGGTTCTATTTTGTTGGTGTTCGGGACCGAAGTAATGATTGATAGGGACAGTTGGGGGCGTCTGTATTCTGAAGCGAGAGGTGAAATTCTTAGGCCTTCGGAAGACGAACAGCTGCGAAAGCGTTCGCCAAGGATGTTCCCTTTGATCAAGAACGAAAGTTGGGGGATCG | 50.81 |
| denovo56 | AGCCGCGGTAATTCCAGCTCCAATAGCGTATATTAAAATTGTTGCGGTTAAAACGCTCGTAGTTGGATGTATGCGAATGAATTTCGGTCCTTCCCAGAGAAGAATACACGGGATTTCATTTGTATTTGTTCAGGGATGGTGGATGCACTTCACTGTGCGACCACATAATCTGGATTTTTACTTTGAGGAAATAAGAGTGTTCCAAGCAGGCAGTTGCCGTGAATATGATAGCATGGAATAATAGTACAAGACTCTTGGTTCAATGCTGTTGGTTTGTTGAGCTAGAGTAATCAAAATAAGGATAGTTGGGGGTATTCGTATTTAACTGTCAGAGGTGAAATTCTTGGATTTTTTAAAGACGAACAATTGCGAAGGCATCTACCCAGGATGTTTTTGTTGATCAAGAACGAAAGTTAGGGGATCG | 40.33 |
| denovo57 | AGCCGCGGTAATTCCAGCTCCAATAGCGTATATTAGAGTTGTTGCAGTCAAAAAGCTCGTAGTTGAATTTCTGCTGGGCCGAACTGGTCCGCCTTCGGGCGTGCACCTGCCAGTCCC-AGCGTTTTTCCCGGTTCCCCGGTGT--TTTA-T-TACGCCGGTTCTCCGGGGACGTTTACTTTGAGAAAAATAGAGTGTTTCAAGCAGGCTATCGCCTTGAATACTGCAGCATGGAATAATAATATAGGACTTTGGCCCTATTTTGTTGGTTCTAGGACTAAAGTAATGATTAATAGGGACAGTTGGGGGCATTCGTATTTAAATGTCAGAGGTGAAATTCTTAGATTACTTAAAGACGCACTAGTGCGAAAGCATTTGCCAAGGATGTTTTCATTAATCAAGAACGACAGTAGGGGGTT | 44.26 |
| denovo63 | CGGTAATTCCAGCTCCAATAGCGTATATTAAAGTTGTTGCGGTTAAAACGCTCGTAGTTGGGTTTCAGGCAGGGTCGTTCGGTCTGTCCTGAGTGTCAAGTCACCGGATCGGCCCTGTCATCTTCTTGGGGAACATGTGCGCACTTCACTGTGCGGCATGGAAACCTTGACATTTACTTTGAGGAAATGAGAGTGCTTCAAGCGGGTATCCGCCACGAATATGTTAGCATGGAATAATAGCATTGGACGTCGAATCGTAGCTGTTGGTTGCTCGGATCGACGTGAGGGTAAACAGGGACAGTTGGGGGCATTCGTATTTAACTGTCAGAGGTGAAATTCTTGGATTTGTTAAAGACGGACTACTGCGAAAGCATTTGCCAAGGATGTTTTCATTGATCAAGAACGAAAGTTAGGGGATCG | 47.14 |
| denovo65 | AGCCGCGGTAATCCCAGCTCCAGTAGCGTATATTAAAATTGTTGCGGTTAAAACGCTCGTAGTTGGACATTTGTAGGAGACTGCCGGTCTACCCCAGTGGTCAGTACTTGGCATGTCTCTTGCACCTTCCTGGTGGAAGTGTTCGTACTTAATTGTGCGGGGCGTAATCCAGAACTTTTACTTTGAGGAAATGAGAGTGCTTCAAGCAGGCATCCGTCGTGAATACGTTAGCATGGAATAACACTATAGACCACGTTCTAGAGCTGTTGGTTTTTAGGACGAGGTAATGAAAAGAGGGATAGTTGGGGGCATTCGTATTTAACTGTCAGAGGTGAAATTCTTGGATCTTTAAAAGACGAACTACTGCGAAAGCATCTGCCCAGGATGTTTCTATTAATCAAGAACGAAAGTTAGGGGATCG | 44.89 |
| denovo66 | AGCCGCGGTAATTCCAGCTCCAATAGCGTATATTAAAGTTGTTGTGGTTAAAAAGCTCGTAGTTGGATCTCGGCGGTCTGAGGGCGGTTCACTTGCTAGTGCTACTGCCCTATAGACCGTAGTTTTGCCAGAGGTCTCAGGGTGCTCTTAATCGAGTGTCTTGTGATGCTGGCAGGTTTACTTTGAAAAAATTAGAGTGCTCAAAGCAGGCTTTTACGCCTGAATATTCGTGCATGGAATAATAGAATAGGAAGTCGTTTCTATTTTGTTGGTTTTCGGAAATCGACTTAATGATTAATAGGGACAGTCGGGGGCATTCGTATTCAAACGACAGAGGTGAAATTCTTGGACCGTCGCAAGACGAACTACTGCGAAAGCATTTGCCAAGGATGTTTTCATTAATCAAGAACGAAAGTTAGAGGTTCG | 43.66 |
| denovo7 | CGGTAATTCCAGCTCCAGTAGCGTATATTAAAGTTGTTGCGGTTAAAACGCTCGTAGTTGGGTTTCAGGCAGGGTCGTTCGGTCTGTCCTGAGTGTCAAGTCACCGGATCGGCCCTGTCATCTTCTTGGGGAACAGGTGCGCACTTCACTGTGCGGCCTGGAAACCTTGACATTTACTTTGAGGAAATGAGAGTGCTTCAAGCGGGTATCCGCCACGAATATGTTAGCATGGAATAATAGCATTGGACGTCGAACCGTAGCTGTTGGTTGCTCGGATCGGGGTGAGGGTAAACAGGGACAGTTGGGGGCATTCGTATTTAACTGTCAGAGGTGAAATTCTTGGATTTGTTAAAGACGGACTACTGCGAAAGCATTTGCCAAGGATGTTTTCATTGATCAAGAACGAAAGTTAGGGGATCG | 48.33 |
| denovo73 | AGCCGCGGTAATTCCAGCTCCAATAGCGTATATTAAAATTGTTGCGGTTAAAACGCTCGTAGTTGGATATCTGCTAAGGGGTTCCGGTCCTTCCCAGTGAAGAATACGCGGAACTCTTCTTGGCATTTATTCAGGGAAGGTGTTCGCACTTTGTTGTGTGTCACGTGATCTGAATTTTTACTTTGAGGAAATGAGAGTGTTTCAAGCAGGCTTTCGCCGTGAATATGATAGCATGGAATAATAGCACAGGACCCCTTTCCAAAGCTGTTGGTTTTTGGAACGAGGTAATCAGAATAAGGATAGTTGGGGGTATTCGTATTTAACTGTCAGAGGTGAAATTCTTGGATTTTTTAAAGACGAACTATTGCGAAGGCATCTGCCCAGGATGTTTTTATTGATCAAGAACGAAAGTTAGGGGATCG | 42.42 |
| denovo75 | CGGTAATTCCAGCTCCAGTAGCGTATATTAAAGTTGTTGCGGTTAAAACGCTCGTAGTTGGGTTTCAGGCAGGGTCGTTCGGTCTGTCCTGAGTGTCAAGTCACCGGATCGGCCCTGTCATCTTCTTGGGGAACAGGTGCGCACTTCACTGTGCGGCCTGGAAACCTTGACATTTACTTTGAGGAAATGAGAGTGCTTCAAGCGGGTATCCGCCACGAATATGTTAGCATGGAATAATAGCATTGGACGTCGAACCGTAGCTGTTGGTTGCTCGGATCGGGGTGAGGGTAAACAGGGACAGTTGGGGGCATTCGTATTTAACTGTCAGAGGTGAAATTCTTGGATTTGTTAAAGACGGACTACTGCGAAAGCATTTGCCAAGGATGTTTTCATTAATCAAGAACGAAAGTTAGAGGTTCG | 47.86 |
| denovo79 | AGCCGCGGTAATTCCAGCTCCAATAGCGTATACTAAAGTTGTTGCAGTTAAAAAGCTCGTAGTTGGATTTCGGGTTGGGCGTGGTCGGTCAACCGCAAGGTTTGTCACTGGCCGGGCCTTCCTTACTCGCGAAGACATCGTGTGCTCTTCACTGAGTGTGCGGTGGATTTGCGATGTTTACTTTGAAAAAATTAGAGTGTTCAAAGCAGGCCGAT-TGCTTGAATGCGTAAGCATGGAATAATGGAATAGGACTTTGGTTCTATTTTGTTGGTTTTCGGAGCCGAAGTAATGATTGACAGAAATGATTGGGGGCATTCGTATTTCGCTGTCAGAGGTGAAATTCTTGGACCTGCGAAAGACGAACTAATGCGAAAGCATTTGTCAAGAGTGTTTTCGTTGATCAAGAACGAAAGTTAGAGGATCG | 44.94 |
| denovo94 | AGCCGCGGTAATTCCAGCTCCAATAGCGTATATTAAAGTTGTTGTGGTTAAAAAGCTCGTAGTTGGATCTCGGCGGTCTGAGGGCGGTTCACTTGCTAGTGCTACTGCCCTATAGACCGTAGTTTTGCCAGAGGTCTCAGGGTGCTCTTAATCGAGTGTCTTGTGATGCTGGCAGGTTTACTTTGAAAAAATTAGAGTGCTCAAAGCAGGCTTTTACGCCTGAATATTCGTGCATGGAATAATAGAATAGGAAGTCGTTTCTATTTTGTTGGTTTTCGGAAATCGACTTAATGATTAATAGGGACAGGCGGGGGCATTCGTATTGCGACGCTAGAGGTGAAATTCTTGGACCGTCGCAAGACGAACTACTGCGAAAGCATTTGCCAAGGATGTTTTCATTAATCAAGAACGAAAGTTAGAGGTTCG | 44.37 |
| denovo96 | AGCCGCGGTAATTCCAGCTCCAATAGCGTATATTAGAGTTGTTGCAGTTAAAAAGCTCGTAGTTGGATTTCTGCTGGG--CA---AGACCGGTGATTAATC----T-CTCCGCGTTTACCCCAGCACTTTCCCCG--GCTATTGCGGCA---TACGCCGT-GATATC-CA----CGGGACCTTTACTTTGAGAAAAATAGAGTGTTTCAAGCAGGCTTTACGCCTTGGATACTGCAGCATGGAATAATAAGATAGGACTTTGGTTCTATTTTGTTGGTTA-TAGGACTAAAGTAATGATTAATAGGGACAGTTGGGGGCATTCGTATTCAACAGTCAGAGGTGAAATTCTTAGATTTGTTAAAGACGAACTACTGCGAAGGCATTTGCCAAGGATGTTTTCATTAATCAAGAACGAAAGT | 40.24 |
